# Tubercular Tumour of the Vertebræ Opening into the Œsophagus

**Published:** 1847-10

**Authors:** 


*Tubercular Tumour of the Vertebræ opening into the Œsophagus.*—A female, aged 29, entered the hospital of Bassano for an obscure affection, accompanied by extreme marasmus, which had supervened upon her last confinement. She had very great difficulty of swallowing, repeated vomiting, difficulty of breathing, and great general debility. She died completely exhausted by hectic fever. On examination after death both pleuræ were found adherent, and behind them, directly over the vertebral column, a tumour was discovered, about the size of a walnut, and springing from the fifth dorsal vertebræ. A second tumour, of larger size, was also seen to include the bodies of the fourth, fifth, and sixth vertebræ, the osseous structure of which was converted into a soft caseous matter. On opening the œsophagus, that canal was found to be narrowed, and firmly adherent to the most prominent part of the last-mentioned tumour, a portion of the contents of which had escaped through an irregular ulceration of bad aspect.—*Prov. Med. and Surg. Journal, from Giornale dei Progressi.*

